# Riggs's Disease—What Is It, and Can It Be Cured?

**Published:** 1894-01

**Authors:** G. A. Mills

**Affiliations:** New York.


ARTICLE III.

### RIGGS'S DISEASE—WHAT IS IT, AND CAN IT BE CURED?

BY DR. G. A. MILLS, NEW YORK.

Some thirty years have elapsed since Dr. Riggs made known his views concerning prevalent disordered conditions of the teeth and their surrounding tissues, and his mode of dealing with them. It would be a waste of time to discuss the general notice and attention given to this before Dr. Riggs gave expression to his views.

This was the common remark, "It is evidence of senility that such conditions are made possible, and there is no remedy." If it had been said that these disordered conditions evidenced an atrophy or hypertrophy, then there might have come the thought of a rationale, for the term senility was only applied to advanced years. It can not be admitted for a moment that no such disordered conditions could be found among the middle or even those nearer the senile period. Loosening and falling out of teeth were confined mostly to the advanced stage of the disorder; the early manifestations did not attract the attention now given them.

When Dr. Riggs first began his teachings, he spoke by the light he then possessed, and that is all any one can do. His light was far in advance of those who had spoken before him. The views expressed were at once regarded as not only new but novel.

Dr. Riggs had the advantage of the light of genius, fortified by a classical education. Genius has the virtue of integrity coupled with this light; they are gifts; keep them harnessed together and that which is derived from observation and experience will be the truth. With such helpers the doctor applied his observation to these generally neglected conditions, and found that there was a disease located about the sockets of the teeth, resulting in pus discharges, loosening of the teeth, and ultimately a loss of attachment of surrounding tissues, falling out of the teeth, and more often than otherwise he found a calcareous deposit, popularly known as tartar.

He was led to go farther in his operations, that he might find the existing cause, at least, of the pus present. Further research was made for tracing out the source of this, that either recommenced after the partial treatment or might have still continued to flow. Patient labor revealed the fact that the alveolus had become a participant of the inflammatory parts, wasted frequently in portions, and sometimes as a whole.

Further attention revealed another fact, that the edge of this bone was in a state of partial necrosis. Having the knowledge of surgical principles, he simply supplied them. These are well known to all surgeons,—"cut or trim back of the life line and secure a healthy reaction." Practice proved this in so large a percentage that it is not much of a marvel that the doctor felt that he had discovered something that could become a blessing. Herein lies the merit of the Riggs treatment, emphasized by the unquestioned intelligence of two witnesses,—viz., the late Dr. W. H. Atkinson, of New York, and also Professor Garretson, of Philadelphia.

For several years Dr. Riggs continued to follow out the results of his discovery, for it truly was one. If he had been of the nature of Atkinson, following his inspirations, the world would have had the benefits sooner. Although it has come slowly to our ears, it has proven that "slow growth is sure." I have said that Dr. Riggs followed the light he had. If we have done as well, then well

Dr. Riggs only gave us one published article. This can be found in the extinct publication, the *Pennsylvania Dental Journal.* It was copied in the *Dental Miscellanies.*

I think this article can be found in the numbers of 1877. That this subject has not been understood is shown by reference to our literature. Very few have become proficient in dealing with this disorder by the method taught by the originator.

It is true that he committed himself to the theory that tartar was the cause,—I would say the exciting cause,—and too many have followed in his thought, and have taken narrow views of the disorder because of this, and have tried to confine his labors to a very limited field, separating salivary deposits from manifestations following the hidden accretions, and neglecting the disordered edge of the alveolus.

This is only an evidence that they have listened with their ears but have failed to understand the teachings. I am safe in this statement, for many have put themselves on record proving the truth of the assertion. The larger proportion of men who have given expressions of their views have ignored the merits of Dr. Riggs' treatment. The mass of instruments that have been put on the market prove it by their forms.

I trust I have now made myself clear what this disease means from a practical standpoint and as announced by Dr. Riggs. It will be noticed that I adhere to the term "Riggs' disease;" this is only to keep the association for a time longer, while the settlement shall be made upon the most intelligent term better expressing the real meaning.

This term did not originate with Dr. Riggs or myself, but with the Connecticut Valley Dental Association. This may appease some. I will add this question: Is it true or not that it is essential that this disorder must be met successfully by observing the merits of the Riggs treatment,—trimming the disordered edge of the alveolus? This is the point at issue. From a large observation I am convinced that ninety-five per cent. are dealing with this disease apart from the recognition of this claimed merit.

Many, a great many, are not observing even the necessity of anything but local dressings; no surgical instruments of any form are used. What does this prove? Just this, the "partial culture" of our calling. It also proves another thing,—that the intelligence has not dawned upon these minds that this disorder has its origin outside of the local conditions; a lack of knowledge to discern the undeniable evidences that this lesion is of constitutional origin. Do many who deny this fact realize that the statements that are so freely and fulsomely made in every direction that you cannot cure pyorrhœa is a proof that it is of constitutional origin.

I can only, from a common-sense view, deny that pyorrhœa is a disease. It is an adjective when used in connection with alveolaris. And if it did express the meaning of a disorder, it is far from covering the range of its destruction in its application to the waste of gum and other tissues that are the normal support of the teeth.

Dr. Rehwinkle admitted to me that this term did not give satisfaction to him. I have always contended against it.

Can Riggs' disease be cured?

If I have made myself intelligent, it will be readily seen that so far as constitutional phases are evidenced in the large majority of cases I can but answer the interrogation in the negative.

No mortal man has the power to cure a disease any more than he can original sin. It can only be arrested or checked by surgical or local treatment, and held in check by constitutional remedies.

Dr. Riggs classed the degrees of progress by numbers 1, 2, 3, 4; this meant to him that number one indicated the beginning, or what I have termed in all my writings and sayings the incipient condition, and number four would mean the last stage, (too late), with which the practitioner is often confronted, for the reason, first that too frequently these incipient conditions were not recognized.

Here a question stares the dentist in the face. Who is to blame for this "too late" stage? Who has been living in darkness in these days of much light? If we assert that this disorder cannot be cured, then what have we to say for all our earnest and zealous labors since 1874? It is this, that intelligence will accomplish much, and if you are able to appreciate the helps offered, you will find after an accumulation of practical experience that you have a stock in trade that will make you effectual in dealing with the needs of your patients. No one thing is so much needed in this day as post-graduate teachings in connection with such a vastly important branch of practice, and one that can be made so fertile with pre-eminent and helpful service for the people. We are in the age of needed demonstration. Our schools are turning out far too many inefficient practitioners that must of necessity prey upon an ignorant public. Our acquirements must be carried upon a higher plane if we are to be just to our patients. Many grave questions are looking in our face. Same one, perhaps more than one, will ask, Will you give a *resume* of our nearly eighteen years' practical experience with this disorder? This can be done with but little effect except by clinical demonstration, case after case. Some will possibly say that this is a disorder which cannot be cured.

What is the encouragement? Not a few laymen are asking the same question. One; yesterday, an intelligent lawyer, came in for counsel. He has had his case treated for pyorrhœa with aromatic sulphuric acid by an M. D. Dentist. The parts are full of pus again, after a few months' treatment. He asked, "Is there any such disease as pyorrhœa?" He was told plainly and decidedly that there was not, and then was given an intelligent statement of facts. He followed by asking another question: "If it cannot be cured, then why not have the teeth extracted?" I said to him, "The people that seek my service are desirous of trying to keep their teeth. I am doing what an intelligent practitioner can to give you the advantage of that which has been gained by years of earnest special practice." There is no nobler nor more humane field of labor to which one can devote himself as a special life-work. The field for this is immense, and there are yet very few who are able to meet the demands. It has not been specialized to any extent, but is largely generalized with very weak dealing. Teeth cannot be saved, except in a minimum percentage of cases, without giving them the best possible surroundings of health.

The practitioner, to meet this demand, must be able to labor in this field with such a degree of culture that the medical practitioner will be compelled to surrender to the superior skill. What men are best fitted to do by nature's endowments should receive special education for special service. This must and will come, for such a skill will ultimately receive a compensation worthy of its hire. Nothing is more true than, as Professor Taft said at one of the late meetings of our profession, "When you undertake to deal with one of these cases of pyorrhœa you must look at the bodily conditions, and find out what you have to enable you to hold the benefits secured by surgical dealings and constitutional treatment." I have given the substance of his remarks. I could detail case after case, but give one where, during the last year and a half, it has been almost entirely dealt with by constitutional remedies.

There were no "calcic" deposits, as Professor Pierce terms them, but decided portions of necrosed bone, and most of the teeth hanging loosely. I had, unexpectedly, an opportunity of showing this case to four dentists, and was asked what I expected to accomplish. I replied, "I don't know, for I never had a case with such peculiar complications of bodily ailments and the patient as young," —only about thirty years of age. It is a case of interest now, and is, in the main, a success,—not complete in all things, but enough so to feel joyful over, considering the painful condition of the teeth and surrounding tissues and also the wrecked physical organization, occupied with serious mental complications. I did not intend to give a *resume* of my treatment, but have presented the subject as I have, hoping to stir a larger intellectual thought upon a matter that is worthy.—*International Dental Journal.*

